# A Journey of Hope: giving research participants a voice to share their experiences and improve community engagement around advanced HIV disease in Uganda

**DOI:** 10.12688/aasopenres.13104.2

**Published:** 2020-10-29

**Authors:** Fiona V. Cresswell, John Kasibante, Emily M. Martyn, Lillian Tugume, Gavin Stead, Kenneth Ssembambulidde, Morris K. Rutakingirwa, Enock Kagimu, Laura Nsangi, Carol Namuju, Jane F. Ndyetukira, Cynthia Ahimbisibwe, Florence Kugonza, Alisat Sadiq, Alice Namudde, Joanna Dobbin, Diksha Srishyla, Carson Quinn, Mable Kabahubya, Conrad Muzoora, Stephen Watiti, David B. Meya, Alison M. Elliott

**Affiliations:** 1 Research Department, Infectious Diseases Institute, Kampala, 22418, Uganda; 2 Clinical Research Department, London School of Hygiene and Tropical Medicine, London, WC1E 7HT, UK; 3 MRC UVRI LSHTM Uganda Research Unit, Entebbe, Uganda; 4 Department of Primary Care & Population Health, University College London, London, UK; 5 University of Minnesota, Minneapolis, USA; 6 University of California San Francisco Medical Centre, San Francisco, USA; 7 Mbarara University of Science and Technology, Mbarara, Uganda; 8 Mildmay, Entebbe Road, Kampala, Uganda

**Keywords:** Public engagement, advanced HIV diseases, meningitis, clinical research

## Abstract

Over the last decade excellent progress has been made globally in HIV management thanks to antiretroviral therapy (ART) rollout and international guidelines now recommending immediate initiation of ART in people living with HIV. Despite this, advanced HIV disease (CD4 less than 200 cells/mL) and opportunistic infections remain a persistent challenge and contribute significantly to HIV-associated mortality, which equates to 23,000 deaths in Uganda in 2018 alone. Our Meningitis Research Team based in Uganda is committed to conducting clinical trials to answer important questions regarding diagnostics and management of HIV-associated opportunistic infections, including tuberculosis and cryptococcal meningitis. However, clinical research is impossible without research participants and results are meaningless unless they are translated into benefits for those affected by the disease. Therefore, we held a series of community engagement events with the aims of 1) giving research participants a voice to share their experiences of clinical research and messages of hope around advanced HIV disease with the community, 2) dispelling myths and stigma around HIV, and 3) raising awareness about the complications of advanced HIV disease and local clinical research and recent scientific advances. The purpose of this Open Letter is to describe our community engagement experience in Uganda, where we aimed to give clinical research participants a greater voice to share their experiences. These activities build upon decades of work in HIV community engagement and lays a platform for future research and engagement activities.

## Introduction

Substantial progress has been made in the treatment and prevention of HIV in the last decade thanks largely to the widespread roll out of antiretroviral treatment (ART) and recommendation to treat all people living with HIV (PLHIV) regardless of CD4 cell count. The ambitious UNAIDS “90-90-90” target states that, by 2020, 90% of PLHIV should be diagnosed, 90% of those diagnosed initiated on ART, and 90% of those on ART should be virally suppressed (HIV viral load <50 copies/ml), aiming to reach the Sustainable Development Goal of ending the HIV epidemic by 2030 ^1^. The global efforts to achieve these targets are demonstrated by a 55% decline of AIDS-related deaths between 2004 and 2018 ^2^.

While unquestionable strides in starting people on ART have been made, gains in recent years are decreasing and 23,000 people died from HIV-related illness in 2018 in Uganda alone ^2^. This is in part due to the remaining challenge of advanced HIV disease, defined by the World Health Organisation (WHO) as having as CD4 cell count less than 200 cell/µL or clinical stage III or IV disease ^3^. Uganda is making good progress towards the “90-90-90” targets, with 84% aware of their HIV status, 87% of those who are HIV positive on treatment and 88% of those on treatment virally suppressed, however, stigma still acts as a barrier to seeking HIV testing and care ^4^. Moreover, those who successfully test may not link to care early enough due to systemic barriers. Data suggest that in sub-Saharan Africa at least one-third of people starting on ART present with advanced HIV disease, a fact which requires addressing if global targets are to be reached ^5^.

Our Meningitis Clinical Research Team based at the Infectious Diseases Institute (IDI) Kampala and Mbarara Regional Referral Hospital Uganda, is dedicated to reducing advanced HIV-associated mortality by improving the diagnosis and treatment of common opportunistic infections, including cryptococcal and tuberculosis meningitis. Together cryptococcal disease and tuberculosis cause over half of HIV-related deaths ^6, 7^.

**Figure 1.**
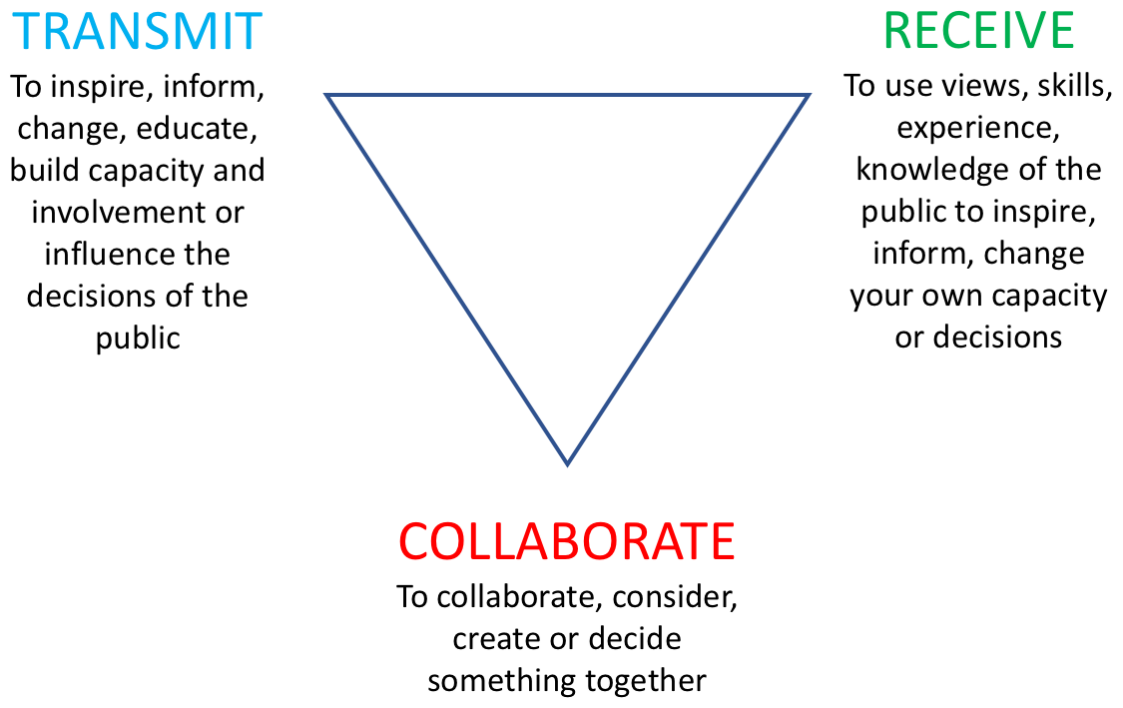
Purposes and pillars of public engagement.

Public engagement seeks to overcome the disconnect between scientists and the community, making research more meaningful for the public and scientists alike. It can serve to improve uptake of research studies and can tackle suspicion about the intention of scientists. Three broad and often overlapping purposes (or pillars) of public engagement are: 1) to ‘transmit’ in order to inspire and inform, change, educate, build capacity and involvement, influence decisions; 2) to ‘collaborate’ in order to consider, create or decide something together; 3) to ‘receive’ in order to use the views, skills, experience of the public to inspire and inform our own capacity ( Figure 1) ^8^. The specific roles of the Meningitis Clinical Research Team in ‘transmitting’, ‘receiving’ and ‘collaborating’ varied according to the type of outreach activity.

Globally, there is a strong history of community stakeholder engagement and activism among PLHIV. This is particularly true in Uganda, where community engagement has long been a priority of civil society groups, such as The AIDS Support Organisation (TASO) based on the Greater Involvement of People Living with HIV/AIDS (GIPA) principles ^9^. We aimed to build on these efforts and also believe it is critical to ensure research participants have a voice to share their experiences and an opportunity to become advocates of clinical research and the condition being studied; their messages may improve the wellbeing and save the lives of others in their communities. With the support of public engagement funding through London School of Hygiene and Tropical Medicine and Makerere University/Uganda Virus Research Institute Infection and Immunity Centre of Excellence, we designed and implemented a series of multifaceted events between December 2018 and January 2020. The overarching aims included giving clinical research participants (who are survivors of advanced HIV disease) a voice in sharing their experiences of clinical research and messages of hope around advanced HIV disease with the community. This included dispelling myths and stigma around HIV, raising awareness about local ongoing clinical research in the field and recent scientific advances. We addressed our aims by engaging a variety of audiences including healthcare workers through knowledge exchange sessions, and thereafter the community and stakeholders in the HIV field through community advisory board meetings, radio, television and two community events. In this letter, using the three pillars of public engagement (transmit, collaborate and receive), we discuss the planning, conduct and outcomes of our public engagement events.

### Knowledge exchange with district healthcare workers. Audience: Health care workers. Purpose: Transmit, receive and collaborate

**Figure 2.**
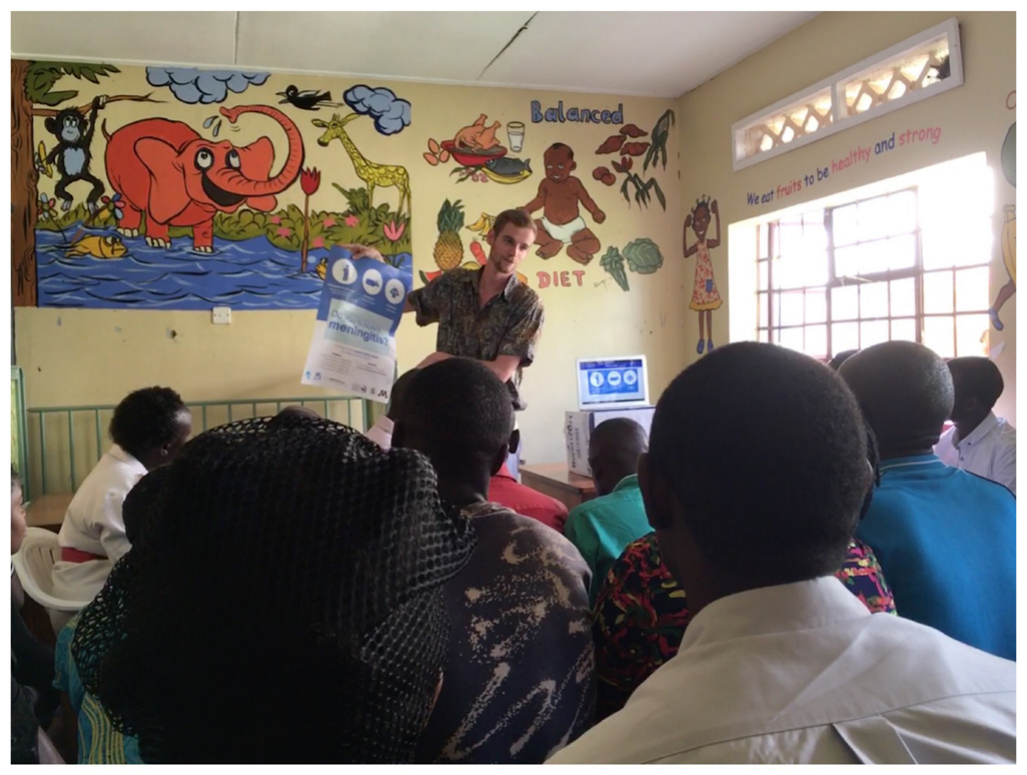
Knowledge exchange session in Western Uganda.

**Figure 3.**
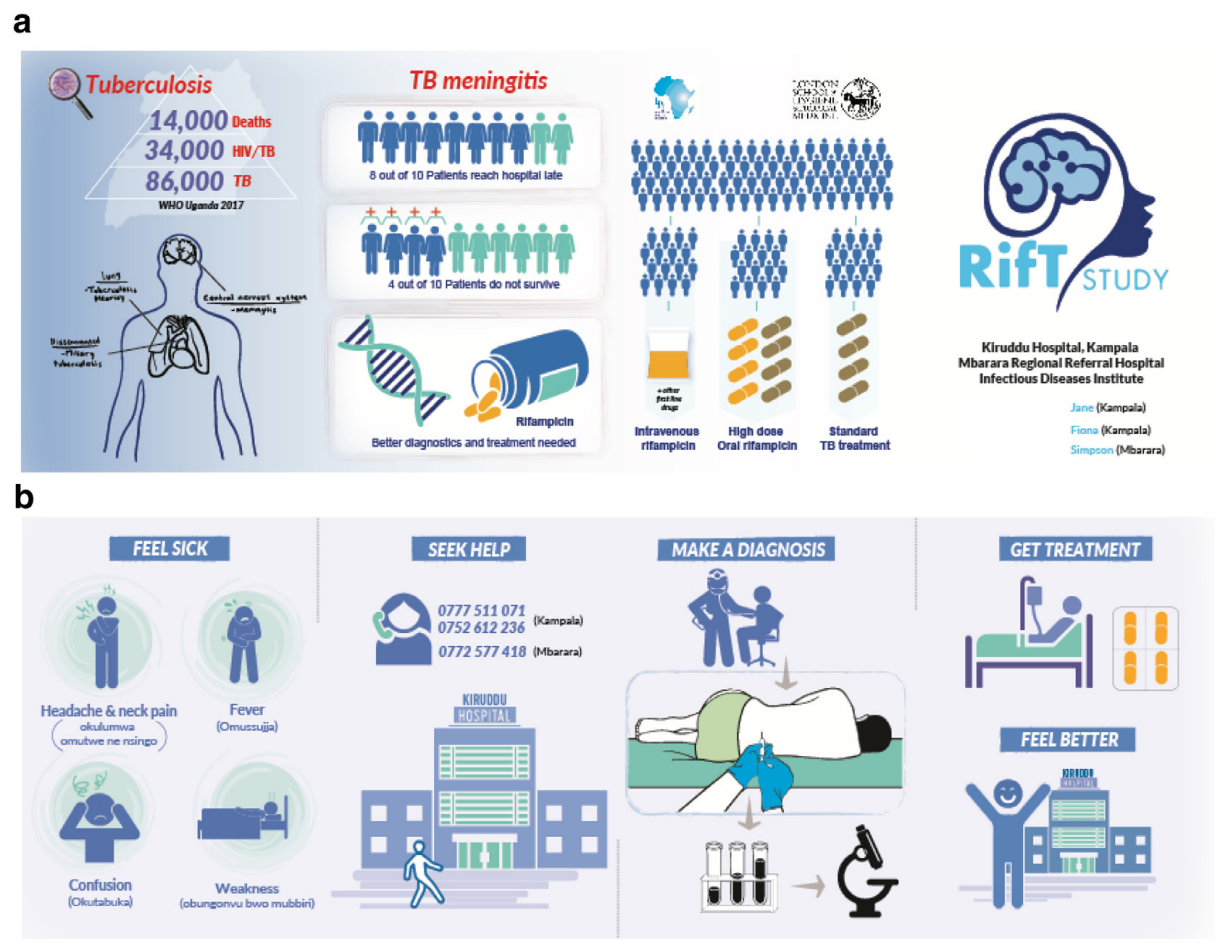
Infographic leaflets distributed to share information about TB meningitis and the RifT clinical trial. The RifT clinical trial studied the safety, tolerability and pharmacokinetics of high dose rifampicin compared to standard of care TBtreatment of adult tuberculous meningitis in Uganda.

For PLHIV presenting with symptoms of meningitis or low CD4+ T cell count, the WHO recommends point-of-care cryptococcal antigen testing (a rapid test for a fungal infection, which causes meningitis in immunocompromised people). However, we noted that most patients were referred on to Kiruddu and Mbarara referral hospitals at a late stage of illness, without prior lumbar puncture (a clinical procedure performed to collect cerebrospinal fluid via a needle placed in the base of the back which is essential for diagnosing meningitis, also known as ‘spinal tap’) or cryptococcal antigen testing ^3^. We visited 21 peripheral health centres in Kampala and 4 centres in Mbarara within the catchment area for Kiruddu and Mbarara hospitals respectively. These were mainly level IV healthcare facilities (HCIV) or district hospitals offering HIV prevention, care and treatment services to HIV-positive clients. In these interactive ‘knowledge exchange’ sessions the meningitis team had three aims. We transmitted information to the healthcare workers using posters, infographic leaflets and power point presentations detailing the aetiology, pathogenesis, clinical presentation, diagnostic challenges, available treatment options and opportunities that clinical trials present in improving treatment and care of PLHIV. We also ‘received’ and ‘collaborated’ with healthcare workers by discussing their experiences and challenges managing advanced HIV disease and potential solutions (e.g. helping with patient referrals and diagnostics such as providing CrAg tests) ( Figure 2 and Figure 3).

An average of 40 people attended each session and the audience comprised of healthcare workers including physicians, medical officers, clinical officers, nurses, laboratory personnel, and medical students. The evaluation of these sessions was performed by informal verbal feedback and discussions with staff who attended the teaching sessions. Staff appreciated having up-to-date teaching to ensure they would recognize advanced HIV disease, cryptococcal and tuberculosis meningitis, and felt empowered to refer to the hospital. They also appreciated understanding what resources were available to ensure they could get the best care for their patients. A working relationship was established between the meningitis research team and health workers in the clinics we visited.

### Community and stakeholder engagement

We recognise that challenges in healthcare seeking behaviour in advanced HIV are multifactorial, influenced by policy, healthcare systems, and community cultural, socioeconomic and geographical factors, to name but a few ^10^. We therefore undertook a multifaceted approach to maximise community engagement and information dissemination around the Kampala region as follows.

### *Community Advisory Board Meetings.* Audience: Community representative. Purpose: Receive and collaborate

**Figure 4.**
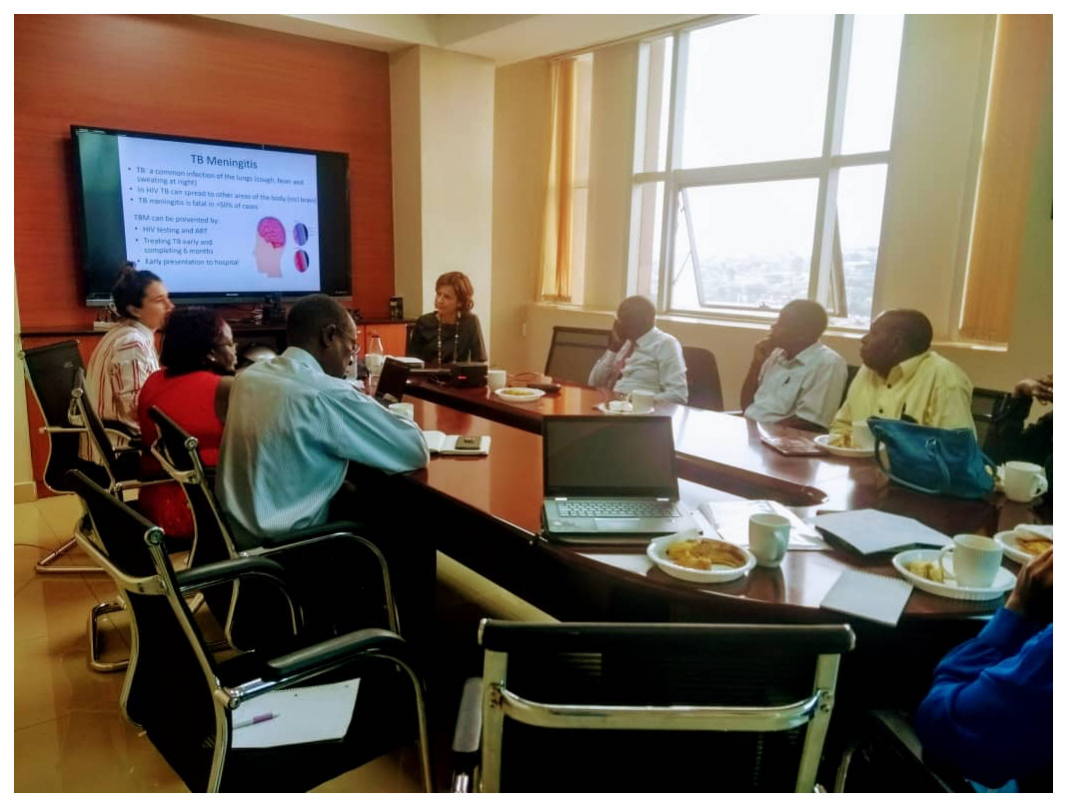
Infectious Diseases Institute Community Advisory Board Meeting.

The IDI Community Advisory Board (CAB; comprised of patient representatives, spiritual leaders, stakeholders and private sector) met with our study team on two occasions to discuss the aims and potential content of our proposed community outreach activities ( Figure 4). Our aim within these meetings was to ‘receive’ opinions and suggestions from the CAB on our proposed outreach activities, and agree appropriate channels of information dissemination and adapting the content and language used to ensure cultural sensitivity Together, we agreed a schedule of events, including a circus event, a television and radio shows, and a ‘Journey of Hope’ event. The members of the CAB also activiely ‘collaborated’ in activities by attending events and a member of the CAB featured in the TV event.

### *Circus event in community center*. Audience: Adults in the community who may be disengaged from HIV care. Purpose: Transmit

**Figure 5.**
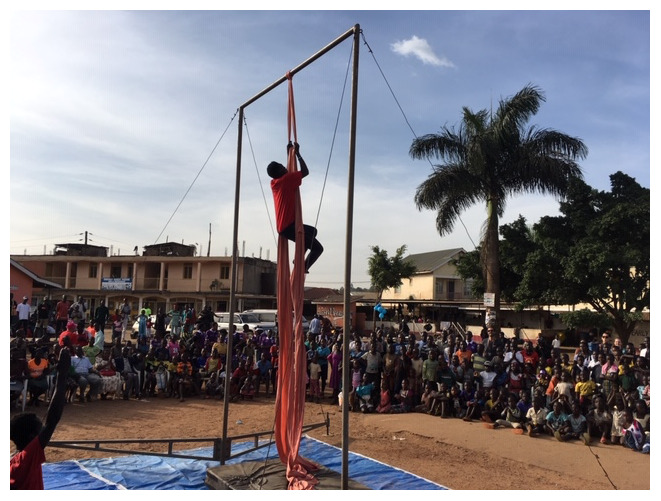
Public audience drawn to the circus event.

In this event the aim of meningitis research team was to transmit: engaging with hard-to-reach young adults who may be disengaged from HIV care or untested in order to raise awareness about meningitis, the safety of lumbar punctures and ongoing meningitis clinical research. Using a local *social circus* group, we attracted a large audience of around 250 people using interactive community performances, music, acrobatics, juggling, fire breathing and a drama sketch of a patient journey through meningitis illness ( Figure 5). A trial participant (Mr JS), Dr Meya (Principal Investigator), Dr Stephen Watiti (an HIV advocate) and a Research Medical Officer did a structured question and answer (Q&A) session with the audience. We conducted formal exit interviews with the audience to ensure that the correct messages had been retained and to receive feedback from attendees. Quotes from exit interviews included *“the event corrected wrong thoughts about meningitis, that it’s a cultural disease or witchcraft”, “the event was very good and it helped people or the community to learn many things in this area”, “Lumbar punctures are not the cause of death in sick people”, “headache, tiredness, neck pain are symptoms of meningitis”.*

### *Radio shows* on *Central Broadcasting Services.* Audience: Community members in central districts of Uganda. Purpose: Receive, transmit and collaborate

**Figure 6.**
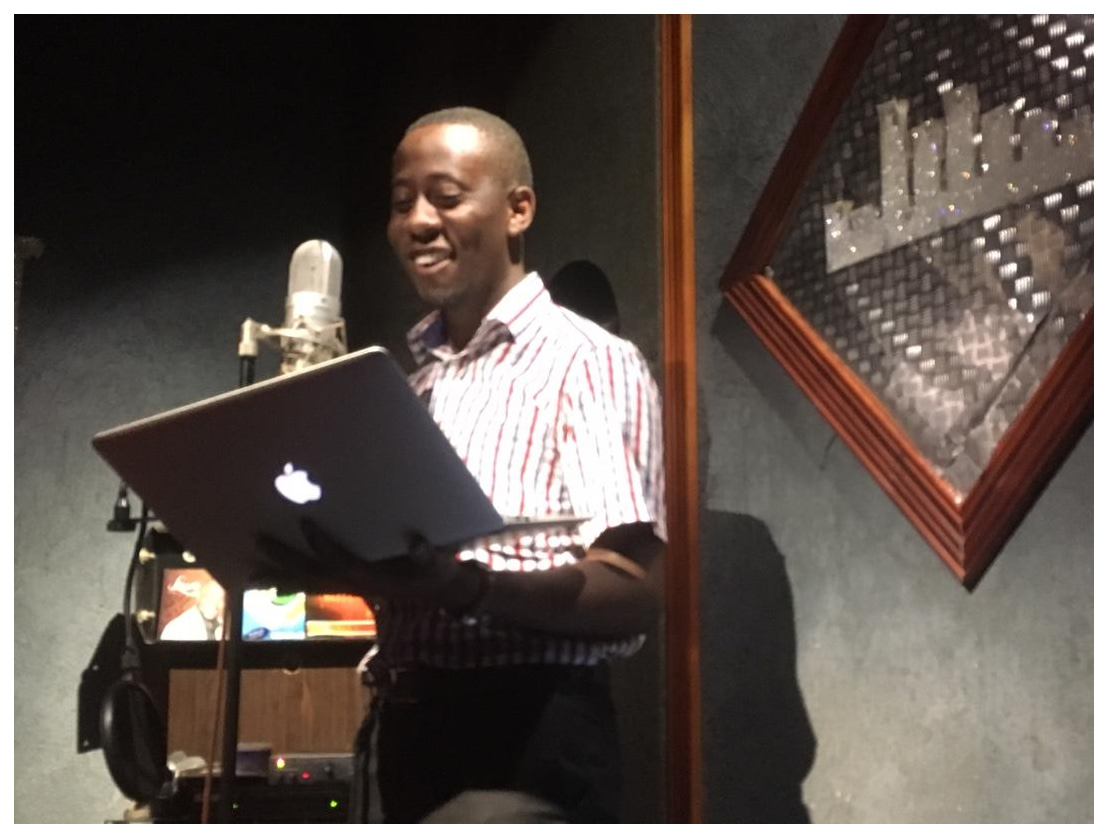
Study physician Dr Ssembambulidde records material for CBS radio broadcasts.

Radio, described as “Africa’s medium of choice in the global age”, remains an important medium of communication in Uganda, in part due to its accessibility ^11^. We harnessed the popularity of radio in attempting to access hard-to-reach populations and aired a series of events on Central Broadcasting Services (CBS) radio, the largest radio station in Uganda. CBS is home to the most popular Breakfast show in Uganda, and a has a large following across different age groups ^12^. The aim of this radio show was to allow the Research Medical Officers to ‘transmit’ important scientific content to the listeners, and also to ‘collaborate’ by co-creating the contents of the radio material with former trial participants, who were able to share their experiences - a focal point of the show. ( Figure 6). In the week leading into World AIDS Day 2019 pre-recorded material including testimonials from three clinical trial participants who have survived advanced HIV disease were broadcast sharing key messages around symptoms of meningitis, experiences with lumbar punctures and clinical research, their treatment and return to health. On World AIDS day itself, a Research Medical Officer on the team featured on the radio and fielded questions from the presenters and public around advanced HIV disease, consistent with the ‘receive’ pillar of the public engagement framework.

### *National Television Show.* Audience: Community who may be disengaged from HIV care or carrying myths or stigma relating to HIV/AIDS. Purpose: Receive , transmit and collaborate

**Figure 7.**
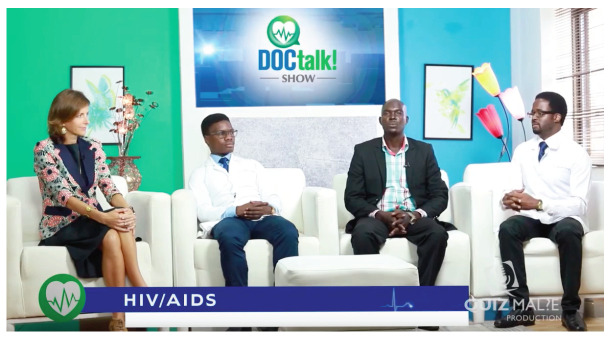
Dr Kasibante, Dr Cresswell and Mr Tugume appear on NTV.

HIV continues to be the most stigmatizing infection in Uganda, in part due to the lack of evidence-based information reaching citizens and long-standing myths regarding HIV/AIDS. Few people with HIV can openly talk about their status and this stigma has led to challenges in reaching the UNAIDS 90-90-90 targets, with 70,000 new HIV infections annually in Uganda and 1 in 3 people presenting with advanced HIV disease ^13^. The key message of this 1-hour television (TV) show, with a wide national audience was that ‘advanced HIV disease is preventable and treatable’. The show was aired on National TV on the World AIDS day 2019 and featured Drs John Kasibante (Research Medical Officer) and Fiona Cresswell (Principal Investigator) and Mr Tugume, a former research participant ( Figure 7). Mr Tugume openly educated people on life as a survivor of advanced HIV disease. He helped demystify key issues such as Undetectable = Untransmissible: early initiation of antiretrovirals to achieve viral suppression and to prevent HIV transmission to loved ones.

During the panel discussion questions arose like “can someone with HIV live with one who doesn’t have HIV and she doesn’t get the infection?”, to which Mr Tugume responded “yes, my wife is HIV-negative, we are raising our son who is also HIV-negative and are soon to have another child”. Mr Tugume also helped to reduce anxiety around lumbar punctures when asked “do people die from lumbar punctures?” and replied “No, I got many lumbar punctures when I was being treated for cryptococcal meningitis in Mulago, and here I am talking with you. They didn’t kill me but saved my life”. This is an example of collaborating with a former research participant to transmit key information to the public. We also received queries and viewpoints from the public.

### *‘A Journey of Hope’ – Research participant and stakeholder event.* Audience: Research participants, institutional leaders, stakeholders. Purpose: Transmit and collaborate

**Figure 8.**
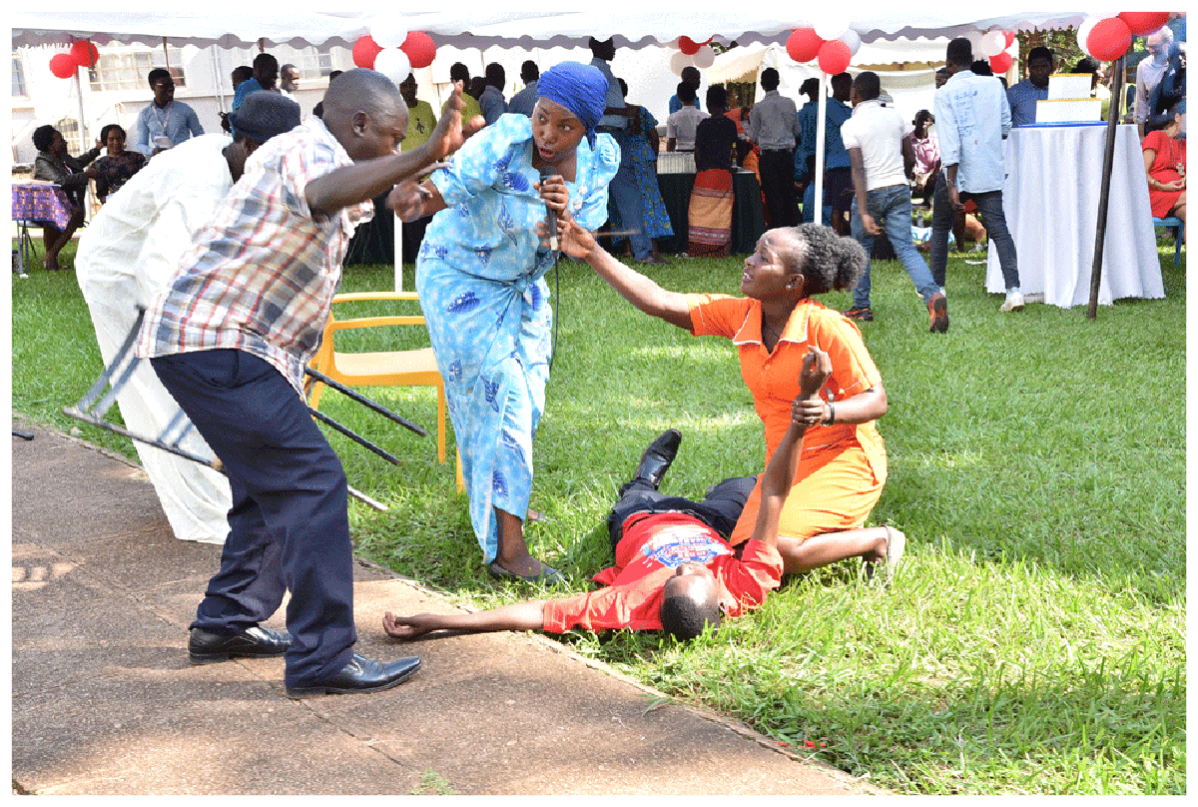
Dramatisation of meningitis illness by IDI drama group.

**Figure 9.**
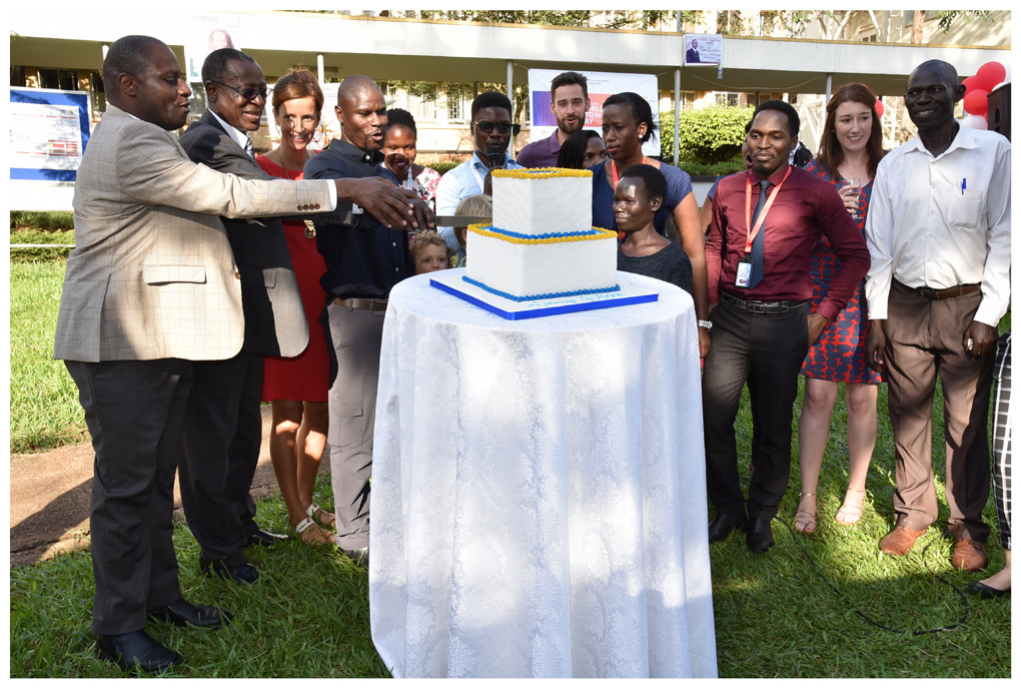
Cake cutting with the research team, study participants and IDI Executive Director.

 ‘A Journey of Hope’ was a celebratory event bringing together clinical trial participants, the IDI Meningitis Research Team, key stakeholders including Centre for Disease Control, U.S. Mission Uganda, Chair of the Mulago Hospital Institutional Review Board, an internationally renowned HIV patient advocate Dr Stephen Watiti, Executive Director of IDI and the research office. The IDI Drama Group performed cultural dances and a dramatization around meningitis ( Figure 8) to address stigma and myths surrounding lumbar punctures and meningitis management (‘collaborate’). A number of trial participants spoke about their experiences in clinical research. The event was also used as a platform to disseminate scientific results to trial participants and important stakeholders (‘transmit’). The ‘Journey of Hope’ symbolised a very difficult journey for patients and their caretakers, many of whom had at times lost hope and were now celebrating with their families and former doctors, empowered to act as community advocates to improve understanding about advanced HIV. Attendees feasted and cut cake together to celebrate the progress made so far ( Figure 9). We hope to repeat this successful event in the future on the completion of further clinical trials.

## Discussion

Whilst it is challenging to measure the exact impact of these activities we know that we engaged with around 500 healthcare workers, 250 members of the public face-to-face, several thousand members of the public through TV and radio, around 80 research participants and a number of key stakeholders in the HIV field. Four former trial participants have come forward as strong advocates for living well with HIV and continue to spread messages of hope in their communities. We learned the importance of harnessing the voices and opinions of the public in planning and conduct of engagement activities and in the planning of future research activities aimed at combating advanced HIV disease. In addition to optimising medical management as stipulated in the WHO advanced HIV care package, we believe addressing late presentation requires a holistic approach, with engagement and education of healthcare workers and the community ^14, 15^.

While there has been a huge amount of positive work in community engagement in PLHIV in Uganda, sometimes public engagement is can be overlooked in academic research. There can be a focus on volume of publications in peer-reviewed scientific journals leading to a lack of value placed on dissemination to the wider public ^16^. However, in an era of digitalisation where ‘fake news’ surreptitiously invades our media sources, it is more important than ever for researchers to take a stand and accurately disseminate research findings to populations who are most affected. As public-researcher interaction improves, so too will trust in science, which may encourage broader public participation in scientific pursuits such as clinical research. We also feel it is critical to give research participants a voice to share their experiences and become advocates of science in the community.

In this letter we have shared our attempt to close the gap between HIV clinical research and the wider public using research participants as partners in delivering a unique variety of novel engagement activities. Our work lays the foundation for future engagement activities and research into the benefits and best practice around public engagement activities.

## Consent

Written informed consent was obtained from all individuals that are identifiable in the provided figures.

## Data availability

### Underlying data

No data are associated with article.
