# Supplementary material for: A Journey of Hope: giving research participants a voice to share their experiences and improve community engagement around advanced HIV disease in Uganda
Source: AAS Open Res. 2020 Oct 29;3:33. Originally published 2020 Jul 27. [Version 2] doi: 10.12688/aasopenres.13104.2 (PMC7682503; doi:10.12688/aasopenres.13104.2)
Supplement: Peer review history

# Peer review: Reviewer response for version 2

**DOI:** 10.21956/aasopenres.14264.r28105

**Published:** 2020-11-20

**Authors:** Deborah Baron

**Affiliations:** 1 Department of Health Behavior, University of North Carolina, Chapel Hill, NC, USA

Thank you again for the opportunity to review this article, and for the revisions made. They are much appreciated, and strengthen the article.

Does the article adequately reference differing views and opinions?

Partly

Are all factual statements correct, and are statements and arguments made adequately supported by citations?

Partly

Is the Open Letter written in accessible language?

Yes

Where applicable, are recommendations and next steps explained clearly for others to follow?

Yes

Is the rationale for the Open Letter provided in sufficient detail?

Yes

Reviewer Expertise:

Good Participatory Practices (GPP) in HIV research; stakeholder and community engagement; participatory and mixed methods research; HIV biomedical prevention research

I confirm that I have read this submission and believe that I have an appropriate level of expertise to confirm that it is of an acceptable scientific standard.

# Peer review: Reviewer response for version 1

**DOI:** 10.21956/aasopenres.14203.r27766

**Published:** 2020-09-21

**Authors:** Deborah Baron

**Affiliations:** 1 Department of Health Behavior, University of North Carolina, Chapel Hill, NC, USA

Thank you for the opportunity to read and review this important Open Letter. The aim of this article was to share how the research team utilized public engagement strategies to: give clinical research participants platforms to share their experiences as research volunteers; dispel myths around HIV and procedures related to advanced HIV disease care and treatment; and raise awareness about their ongoing research studies.

 The Open Letter provides a brief and useful summary of their main engagement activities, as organized around a model of three pillars of public engagement: transmit; receive; and collaborate. These pillars provide a useful framework for the Letter, and adding succinct details on the roles, benefits and challenges faced by the two main implementing partners (i.e. research group and clinical research participants) would further strengthen the Letter. In particular, the inclusion of the ‘audience’ for each sub-section was helpful, and I recommend additionally specifying who is imparting these experiences, skills, and knowledge for each pillar. For example, in Figure 1, for ‘Transmit’ – does the model indicate the research participants are providing the inspiration, and doing the educating and influencing public decisions by sharing their life experiences as part of an advocacy strategy? Or are only the researchers seen as transmitters and the public (which presumably includes study participants) are positioned as receivers?) Were there any challenges in having participants or researchers deliver messages? Clarifying the roles of researchers, study participants and the public as outlined by the model will help the reader better understand the dynamics and added value in delivering these engagement activities in partnership. Please also include a complete citation (#6) in the references for this model.

 Second, it was wonderful reading about the multi-pronged approach and array of creative community-level and media activities undertaken as part of the series. These activities are commendable, and I would encourage the authors to further acknowledge the decades of community stakeholder efforts in the HIV research field that preceded them. While it may be accurate that ‘public engagement is often overlooked in academic research’ broadly, this cannot be said for HIV research specifically. From the Greater Involvement of People Living with HIV/AIDS (GIPA) principles to trail-blazing civil society groups like TASO in Uganda, people living with HIV have always actively demanded their voices be heard and have played crucial roles in contributing to the development and implementation of HIV research, policy, and care and treatment services both globally and in Uganda. This context provides important and differing perspectives; and helps maintain our accountability as researchers to the communities in which we work.

 Further, the Letter makes a strong case for Uganda’s ongoing need to conduct research designed to improve the diagnosis and treatment of common opportunistic infections that contribute to advanced HIV-associated mortality. Minor comments about the research itself that are intended to improve clarity and readability overall include:

- Consider briefly defining the medical term, ‘lumbar puncture’ as this is not common knowledge.
- For Figure 3 – Please clarify if these are actual clinical research recruitment materials that have been approved by a local research ethics committee.
- Recommend adding a line to describe the RIFT Study.
- Request clarification about whether the event, “A Journey of Hope” was a one-off or recurring event? And was it convened around a specific study or a broader research portfolio? The authors note that the event was used to disseminate results, yet also mention they “celebrated the progress made so far” – which implies there is still ongoing research, and thus potentially future events to come? These are important factors to note as they can directly and indirectly impact a group's ability to build trust with the public.

Additional citations, especially where statistics are provided, would strengthen this Letter, and benefit readers. For example, the following sentences ought to include references:

- “Together cryptococcal disease and tuberculosis cause over half of HIV-related deaths.” Pg.3.
- “….70,000 new HIV infection annually in Uganda, and 1 in 3 people presenting with advanced HIV disease” Pg. 6.

Thank you again for sharing your community engagement work with the field. I look forward to seeing how your research and your relationships with the community continue to evolve and strengthen over time as you carry out this important research in reducing advanced HIV disease mortality.

Does the article adequately reference differing views and opinions?

Partly

Are all factual statements correct, and are statements and arguments made adequately supported by citations?

Partly

Is the Open Letter written in accessible language?

Yes

Where applicable, are recommendations and next steps explained clearly for others to follow?

Yes

Is the rationale for the Open Letter provided in sufficient detail?

Yes

Reviewer Expertise:

Good Participatory Practices (GPP) in HIV research; stakeholder and community engagement; participatory and mixed methods research; HIV biomedical prevention research

I confirm that I have read this submission and believe that I have an appropriate level of expertise to confirm that it is of an acceptable scientific standard, however I have significant reservations, as outlined above.

# Response

**Published:** 2020-10-18

**Authors:** fiona cresswell

**Affiliations:** Infectious Diseases Institute, Uganda

*We thank the reviewers for taking the time to review our manuscript, and for their detailed and thought-provoking comments. We have written a point-by-point response for each of the reviewer suggestions.*

*Comment 1. Thank you for your constructive comments. Within the public engagement framework, the pillars of ‘transmit’, ‘receive’ and ‘collaborate’ apply to the meningitis clinical research team. The specific pillar will vary according to the activity in question. For example, at the community advisory board (CAB) meeting the research team are receiving information and opinions from the CAB group and collaborating with the group in order to agree the best program of public outreach activities, taking into account their local expertise and knowledge. However, in the circus event, the research team are predominantly transmitting information to attendees/members of the public. We have now clarified this within the text describing each event.*

*Comment 2. We have now completed the citation *#6 *.*

*Comment 3.* *Thank you for highlighting the important history of public engagement amongst PLHIV and the trail-blazing work of TASO. We have now adjusted our phrasing and specifically acknowledged his in our introduction and discussion, instead focussing on how important public engagement remains in research and how our work builds on the work of our predecessors and colleagues.*

*Comment 4: Thanks for pointing this out, we have now explained the term lumbar punture in parentheses the first time this is mentioned in the text.*

*Comment 5. The infographic leaflets were used along more detailed patient information leaflets, both of which were approved by the local* *research ethics committee*.

*Comment 6. A short description has now been included in the figure legend.*

*Comment 7. The journey of hope was a one-off event at the close of a phase II clinical trial. A phase III trial is just beginning and we hope to continue these events during and at the conclusion of ongoing/further studies.*

Additional citations, especially where statistics are provided, would strengthen this Letter, and benefit readers. For example, the following sentences ought to include references: 

*Comment 8. Thanks for pointing out the missing citations – these have been added.*

*Thank you for taking the time to read, critique and improve this work.*

# Peer review: Reviewer response for version 1

**DOI:** 10.21956/aasopenres.14203.r27765

**Published:** 2020-09-08

**Authors:** Angela Kaida

**Affiliations:** 1 Faculty of Health Sciences, Simon Fraser University, Burnaby, BC, Canada

Thank you for this opportunity to read and review this wonderful Open Letter. The authors describe their process of leading a series of community engagement activities to share clinical research findings with healthcare workers, participants, patients, and the general public. These efforts aimed to give clinical research participants a voice in sharing their experiences of participating in research, further messages of hope around advanced HIV disease, dispel HIV-related myths and stigma, and disseminate recent research findings. I thank the investigators and co-authors for sharing their experiences and insights.

 I request that the authors replace the use of "HIV-positive" terminology with "people living with HIV", consistent with community-based research "people first" language.

 It would be helpful to include additional details about the evaluations undertaken, including whether the evaluation with the healthcare workers was formal (e.g., written anonymous evaluation forms) or informal (e.g., based on discussions held as part of the information session itself) and whether any recommendations were offered for ways to improve public engagement activities.

Does the article adequately reference differing views and opinions?

Yes

Are all factual statements correct, and are statements and arguments made adequately supported by citations?

Yes

Is the Open Letter written in accessible language?

Yes

Where applicable, are recommendations and next steps explained clearly for others to follow?

Yes

Is the rationale for the Open Letter provided in sufficient detail?

Yes

Reviewer Expertise:

HIV, epidemiology, community-based research, women and HIV, sexual and reproductive health

I confirm that I have read this submission and believe that I have an appropriate level of expertise to confirm that it is of an acceptable scientific standard.

# Response

**Published:** 2020-10-18

**Authors:** fiona cresswell

**Affiliations:** Infectious Diseases Institute, Uganda

*Many thanks for your positive feedback on our work and manuscript, gratefully received. We have corrected the language to PLHIV as suggested. Thank you for raising the important point of activity evaluations. The only event which we collected ‘formal’ feedback was for the circus event where exit interviews were conducted. At the healthcare worker ‘knowledge exchange’ events, we only received informal verbal feedback and it was not possible to evaluate the impact of the radio or TB event. In future events we like to create a formal feedback questionnaire so that improvements and suggestions can be documented and acted upon.*
